# Modulating phase segregation during spin-casting of fullerene-based polymer solar-cell thin films upon minor addition of a high-boiling co-solvent

**DOI:** 10.1107/S1600576724010082

**Published:** 2024-11-17

**Authors:** Kuan-Hsun Lu, Wei-Ru Wu, Chun-Jen Su, Po-Wei Yang, Norifumi L. Yamada, Hong-Jun Zhuo, Show-An Chen, Wei-Tsung Chuang, Yi-Kang Lan, An-Chung Su, U-Ser Jeng

**Affiliations:** a Department of Chemical Engineering National Tsing Hua University Hsinchu300044 Taiwan; b National Synchrotron Radiation Research Center Hsinchu300092 Taiwan; c Institute of Materials Structure Science High Energy Accelerator Research Organization Tsukuba Ibaraki305-0801 Japan; d Materials and Electro-Optic Research Division National Chung Shan Institute of Science and Technology Taoyuan325204 Taiwan; e College of Semiconductor Research National Tsing Hua University Hsinchu300044 Taiwan; Argonne National Laboratory, USA

**Keywords:** polymer solar cells, additive effects, spinodal decomposition, X-ray reflectivity, neutron reflectivity, GISAXS, GIWAXS, grazing-incidence small/wide-angle X-ray scattering

## Abstract

The impact of additives on the nanoscale structures of spin-cast polymer composite films, particularly in polymer solar cells, is a topic of significant interest. This study focuses on the blend film comprising poly(thieno[3,4-*b*]thio­phene-*alt*-benzodi­thio­phene) (PTB7) and [6,6]-phenyl-C_71_-butyric acid methyl ester (PC_71_BM), exploring how additives like 1,8-di­iodo­octane (DIO) influence the film structures spin-cast from chloro­benzene solution. Combined results of specular X-ray and neutron reflectivity, grazing-incidence small- and wide-angle X-ray scattering (GISAXS and GIWAXS), and X-ray photoelectron spectroscopy indicate that DIO could significantly enhance the dispersion of PC_71_BM and reduce composition inhomogeneity in the film. Time-resolved GISAXS–GIWAXS with 100 ms resolution further captures a rapid spinodal decomposition of the mixture within 1 s in the constant-evaporation stage of spin-casting. Further combined with parallel analysis of time-resolved UV–Vis reflectance, these findings reveal that DIO mitigates the spinodal decomposition process by accelerating solvent evaporation, which, in turn, decelerates phase segregation, leading to a nucleation-driven process. These observations provide mechanistic insights into the role of additives in controlling the nanostructural evolution of spin-cast films by altering the kinetics of solvent evaporation and phase separation during the spin-coating process.

## Abstract 2

Combined 100 ms-resolved grazing-incidence small/wide-angle X-ray scattering and optical interferometry reveal that the additive diiodooctane can double the solvent evaporation rate, thereby effectively suppressing the rapid spinodal decomposition process in the early stage of spin-coating, favouring slow phase segregation kinetics with nucleation and growth.

## 1. Introduction

Controlled phase segregation in spin-cast blend films is highly relevant for various solution-processed polymer electronic devices, including polymer solar cells (PSCs) and field-effect transistors (Chen *et al.*, 2012 ▸; Liao *et al.*, 2013 ▸; Liu *et al.*, 2013 ▸). Nanoscale segregation between donor conjugate polymers and acceptor fullerene derivatives within the active layers of bulk-heterojunction PSCs is critical for improving device performance. Achieving uniformly intermixed nanodomains of these components enhances exciton generation and creates bi-continuous networks for efficient carrier transport to the electrodes, thereby preventing exciton recombination (van Franeker *et al.*, 2015*a* ▸). Processing methods like spin-casting added to thermal and solvent annealing, along with the use of additives, have significantly increased the power conversion efficiency (PCE) of fullerene-based PSCs to approximately 10% (He *et al.*, 2015 ▸). Meanwhile, developments in all-polymer PSCs have seen PCEs rise to about 18% (Hung *et al.*, 2022 ▸; Xue *et al.*, 2024 ▸); in these developments, additives remain crucial in optimizing film morphology.

In the conventional poly(3-hexyl­thio­phene) and [6,6]-phenyl-C_61_-butyric acid methyl ester (P3HT/PC_61_BM) system, techniques such as post-spin-casting thermal (Wu *et al.*, 2011 ▸; Yang *et al.*, 2005 ▸; Chiu *et al.*, 2008 ▸) and solvent annealing (Shao *et al.*, 2014 ▸; He *et al.*, 2015 ▸) have been effectively utilized to enhance phase segregation of the binary components in PSC active layers. This results in optimized polymer crystalline nanodomains and fullerene nanoaggregates, leading to performance improvements. However, for low-bandgap polymer-based systems like the thieno[3,4-*b*]thio­phene-*alt*-benzodi­thio­phene conjugate copolymer (PTB7) blended with fullerene derivatives like PC_71_BM, additives are crucial to suppress their excessive phase segregation during spin-coating (Liu *et al.*, 2014 ▸; Liu *et al.*, 2015 ▸; Kim *et al.*, 2013 ▸; Lu & Yu, 2014 ▸); thus, nanodomain sizes and connectivity could be optimized for improved charge separation and transport without post-spin-coating processing (Chen *et al.*, 2011 ▸; Wang *et al.*, 2014 ▸; van Franeker *et al.*, 2015*b* ▸; Lou *et al.*, 2011 ▸), as demonstrated through structural probes like grazing-incidence small/wide-angle X-ray scattering (GISAXS/GIWAXS) (Pearson *et al.*, 2013 ▸; Li *et al.*, 2016 ▸; Pröller *et al.*, 2016 ▸; Müller-Buschbaum, 2014 ▸; Hexemer & Müller-Buschbaum, 2015 ▸), depth-resolved X-ray photoemission spectroscopy (XPS) (Wu *et al.*, 2014 ▸), energy-filtered transmission electron microscopy (Rujisamphan *et al.*, 2014 ▸) and neutron reflectivity/X-ray reflectivity (NR/XR) (Kirschner *et al.*, 2012 ▸; Liu *et al.*, 2011 ▸; Wang *et al.*, 2015 ▸). Specifically, in a model blend of PC_71_BM and PTB7, the use of di­iodo­octane (DIO) in chloro­benzene solution for spin-casting significantly boosts the PCE to nearly 10% (He *et al.*, 2015 ▸; He *et al.*, 2012 ▸; Liu *et al.*, 2014 ▸; Wang *et al.*, 2014 ▸; Hedley *et al.*, 2013 ▸). This improvement is attributed to DIO’s low volatility and affinity for PC_71_BM, facilitating a more homogeneous distribution of PTB7 and PC_71_BM across the film thickness. Additionally, DIO has been shown to enhance the dispersion of PCBM nanodomains to approximately 50–100 nm (Jhuo *et al.*, 2016 ▸). Despite the documented effects of DIO on spin-cast PSC films, the mechanisms by which DIO influences phase segregation during spin-casting film formation are still being explored. *In situ* structural observations during spin-casting can offer deeper insights into the role of additives in modulating the nanoscale phase segregation kinetics of the binary components during spin-coating (Wu *et al.*, 2017 ▸).

In this study, we utilize XR/NR, GISAXS/GIWAXS, XPS and atomic force microscopy (AFM) to investigate the PSC active layer structures of PTB7–PC_71_BM. These films were spin-cast from chloro­benzene (CB) solutions, with and without the additive DIO. The structural details were uncovered through analysis of the composition profiles of PC_71_BM and PTB7 across the film’s thickness, via combined NR/XR for contrast variation (Kang *et al.*, 2018 ▸). To gain deeper insights into the phase segregation characteristics observed in these films, we conducted energy pair interaction calculations using Monte Carlo simulations and density functional theory (DFT). Moreover, using 100 ms resolution GISAXS–GIWAXS, we captured a rapid liquid–liquid phase separation (LLPS) in the early stage of the spin-coating process (van Franeker *et al.*, 2015*c* ▸). Time-resolved UV–Vis reflectance analysis further revealed the solvent evaporation features of the solution film during spin-coating. These combined time-resolved measurements shed light on how DIO additive can modify the solvent evaporation kinetics, and therefore the phase segregation kinetics, during the spin-coating process, leading to optimized nanostructures of the PSC films for improved device performance.

## 2. Methods and experiments

### 2.1. Sample preparation

PTB7 was sourced from 1-Materials, while PC_71_BM and 1,8-di­iodo­octane (DIO), both with 99.5% purity, were supplied by Nano-C. The process began by spin-casting poly(3,4-ethylenedioxy­thio­phene) (PEDOT) (Clevios P VP AI4083) onto 14 or 20 mm square silicon wafers, which had been cleaned using oxygen plasma. These films were then dried at 150 °C for 10 min and allowed to stand at room temperature for 20 min inside a glove box. Subsequently, PTB7 and PC_71_BM mixtures, in the optimized ratio of 1:1.5 (*w*/*w*, weight ratio), were prepared in either CB alone or CB:DIO (97:3 *v*/*v*) solutions, at a concentration of 25 mg ml^−1^. These solutions were spin-cast onto the PEDOT/Si films under 1100 rev min^−1^ and then placed in vacuum to deplete residual solvent and DIO in the film. The samples are differentiated by the presence (denoted as D-1.5) or absence (N-1.5) of DIO in the processing solution.

### 2.2. Instrumentation

XR data were measured using a synchrotron 8 keV beam (wavelength λ = 1.55 Å) at the wiggler beamline 17B of the Taiwan Light Source (TLS) of the National Synchrotron Radiation Research Center (NSRRC), Taiwan. NR data were collected at the BL16 SOFIA time-of-flight neutron reflectometer in J-PARC/MLF, Japan (Yamada *et al.*, 2011 ▸). Static and time-resolved GIWAXS and GISAXS were performed at the TLS beamline 23A of NSRRC (Jeng *et al.*, 2010 ▸), with a 10 keV X-ray beam (λ = 1.24 Å) at 0.12°, 0.16° or 0.2° incidence on the sample surface. The setup for an in-line spin-coating system with GISAXS/GIWAXS is shown in Fig. S1 (in the supporting information) and was detailed in a previous report (Wu *et al.*, 2017 ▸). Briefly, 100 µl of sample mixture in CB was dropped onto a silicon wafer (14 mm by 14 mm) pre-calibrated for zero-incidence angle of the X-ray beam on a spin-coater, which was enclosed in an air-tight chamber with Kapton windows for entrance and exit of X-rays. After dropping of the sample solution, the spin-coater was programmed to tilt for 0.2° X-ray beam incidence and ramped up to a speed of 1100 rev min^−1^. Simultaneous GISAXS and GIWAXS data collections were triggered concomitantly with the starting of the spin-coater, which defined the zero-time of the spin-coating process. The GISAXS–GIWAXS data were collected with a resolution of 100 ms to capture the fast spinodal decomposition process over the first 10 s, and with 1 s resolution for the remaining 50 s of the spin-coating process. The evolution of film thickness during spin-coating in the same chamber as used for GISAXS–GIWAXS (as shown in Fig. S1) was monitored at a rate of 4 frames s^−1^ using a UV–Vis reflectance spectrometer (Filmetrics F20-UV) at normal incidence. The film thickness was extracted from the time-resolved reflectance at 632.8 nm, using the procedures reported previously (Wu *et al.*, 2017 ▸; Renaud *et al.*, 2009 ▸; Babonneau, 2010 ▸). Depth-dependent XPS spectra were measured with Ar^+^ etching using a ULVAC-PHI XPS instrument, equipped with a scanning monochromatic Al anode and 180° spherical capacitor analyzer plus 32 channel detectors. The topography and phase images of AFM were taken with a Park System XE-70 instrument. Monte Carlo simulations and DFT calculations (*Dmol3*) within the *Materials Studio* software package (Mayo *et al.*, 1990 ▸; Otto & de Villiers, 2013 ▸) were used to calculate the energy pair interaction among the four components of PCBM, PTB7, DIO and CB.

### 2.3. Data analysis

#### 2.3.1. Contrast variation of XR and NR

Contrast NR and XR datasets of the same sample film were jointly analyzed using the *MOTOFIT* analysis package (Nelson, 2006 ▸), which employs a slab-model approach with the Abeles matrix method for nonlinear least-squares regression. It was used in the co-refinement of consistent X-ray and neutron scattering length density (SLD) profiles, with shared parameters in sublayer thickness and roughness but independent parameters in the neutron and X-ray SLD of the sublayers. The depth-dependent compositions (namely, volume fractions of PTB7 and PC_71_BM and porosity) of the composite films were retrieved from the co-fitted neutron and X-ray SLD profile 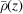 of the sample films following the methodology established previously (Kang *et al.*, 2018 ▸; Liu *et al.*, 2011 ▸) and given briefly in the following. For an *n*-component system, 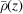
 = 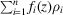 is contributed by the *n* components of the corresponding SLD ρ*_i_*, weighted by the depth-dependent volume fraction *f_i_*(*z*) averaged over the plane at the film depth *z*. Therefore, the two *f_i_*(*z*) profiles of a two-component system can be resolved with the two relationships
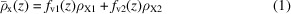
and
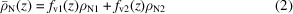
with the porosity volume fraction *f*_v3_ = 1 − *f*_v1_ − *f*_v2_. We note that the X-ray and neutron SLD values of PC_71_BM, ρ_x1_ = 14.5 × 10^−6^ Å^−2^ and ρ_N1_ = 4.97 × 10^−6^ Å^−2^, and those of PTB7, ρ_x2_ = 11.5 × 10^−6^ Å^−2^ and ρ_N2_ = 1.16 × 10^−6^ Å^−2^, are predetermined from independent XR measurements, as shown in Fig. S2.

#### 2.3.2. GISAXS data analysis

Data fitting of GISAXS 1D profiles and 2D pattern simulations were carried out using the *fitGISAXS* software package (Babonneau, 2010 ▸). We note that the 1D GISAXS profile retrieved from either the in-plane or out-of-plane direction of the 2D GISAXS pattern reveals only structural heterogeneity along that direction. Time-dependent scattering invariants 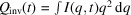 for the GISAXS profiles were calculated using the GISAXS profiles *I*(*q*, *t*) in the *q* range measured, with the scattering vector magnitude *q* = 4πλ^−1^ sin θ defined by the X-ray wavelength λ and scattering angle 2θ.

## 3. Results and discussion

### 3.1. XR and NR for through-thickness composition profiles

**Figure 1:**
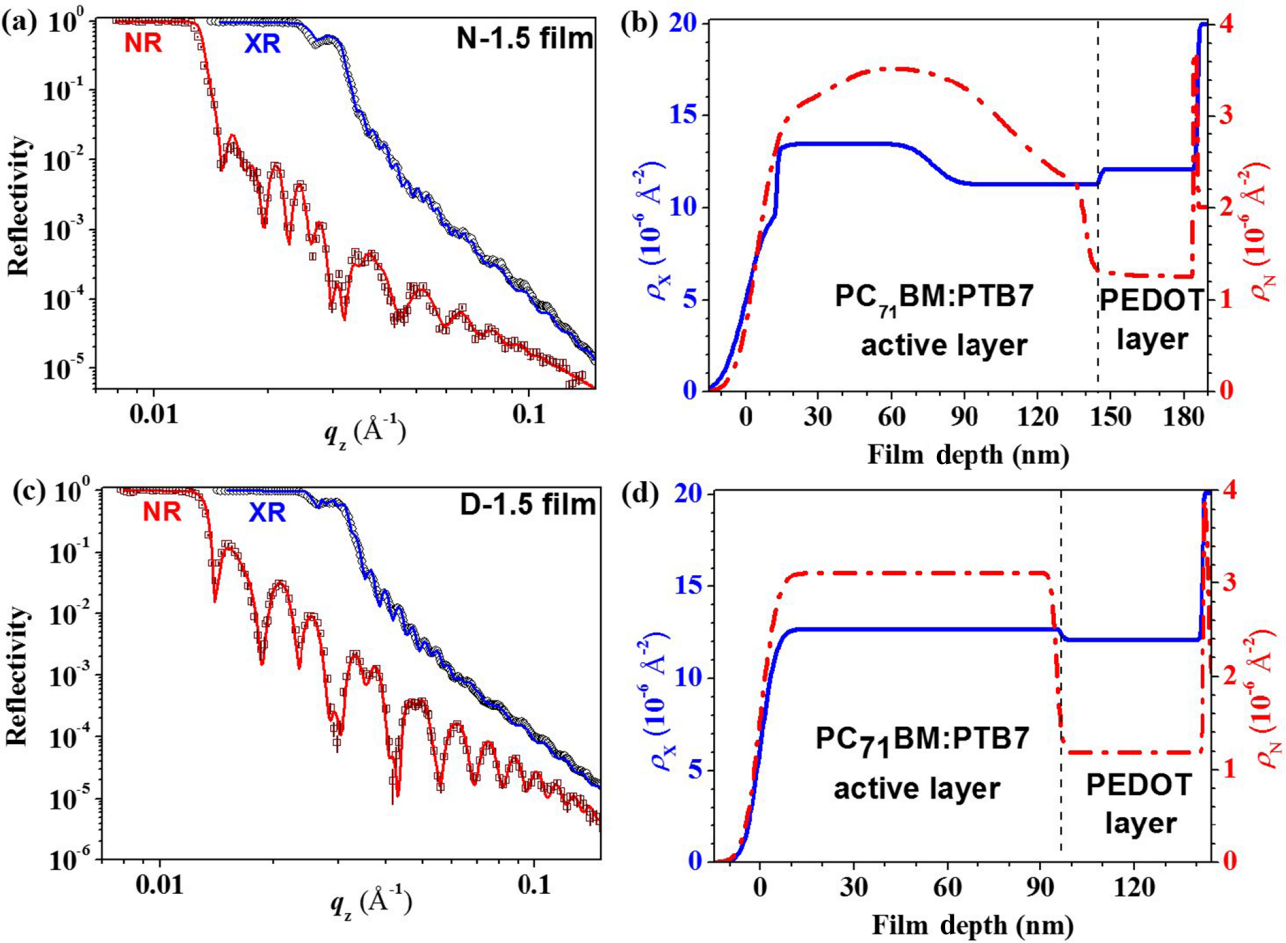
(*a*) XR and NR data fitted (curves) using (*b*) the five-layer SLD models (solid and dashed profiles) for the same N-1.5 film, including the surface sublayer and two main sublayers of the active layer sitting on a PEDOT:PSS conducting layer on the silicon substrate. Note that an additional thin SiO_*x*_ sublayer of *ca* 2 nm thickness above the Si substrate is used in the NR data fitting (which might be produced by UV–ozone treatment on the Si substrate and is sensitive to NR due to its relatively high ρ_N_ value compared with the pure Si substrate); the ρ_x_ value of this SiO_*x*_ sublayer is, however, very close to that of the Si substrate, and therefore the SiO_*x*_ sublayer is neglected in the XR data fitting. (*c*) Corresponding data of the D-1.5 film are fitted using (*d*) the four-layer SLD models. The vertical dashed lines in (*b*) and (*d*) mark the interfaces between the PTB7–PC_71_BM blend layer and the PEDOT:PSS conducting layer.

Fig. 1 ▸(*a*) presents the XR and NR data for the N-1.5 film of PTB7/PC_71_BM (1:1.5 *w*/*w*) processed without the additive DIO. In the reflectivity patterns, Kiessig fringes with two distinct periodicities, Δ*q*_*z*_ ≃ 0.0045 and 0.016 Å^−1^, were identified, corresponding to an overall film thickness of approximately 140 nm and an underlying PEDOT conducting layer of about 40 nm, as estimated using the formula 2π/Δ*q*_*z*_. The XR data, depicted in Fig. 1 ▸(*a*), were optimally fitted using a model that includes the X-ray SLD (ρ_x_) profile shown in Fig. 1 ▸(*b*). This model features a transition surface layer with a low ρ_x_ and a main active layer with fluctuating ρ_x_ values above the homogeneous PEDOT conducting layer. Similarly, the NR data in Fig. 1 ▸(*a*) are best fitted using the corrugated neutron SLD (ρ_N_) profile shown in Fig. 1 ▸(*b*). The enhanced inhomogeneity in the ρ_N_ profile is attributed to a particularly uneven distribution of PC_71_BM along the through-thickness direction. Notably, the NR of the film is predominantly influenced by PC_71_BM, which has a significantly higher ρ_N_ value compared with PTB7.

In the case of the D-1.5 film processed with DIO, the best fitted ρ_x_ and ρ_N_ profiles [Fig. 1 ▸(*d*)] from the XR/NR contrast data set shown in Fig. 1 ▸(*c*) reveal significantly smoother profiles compared with those for the films processed without DIO. The fitted values of ρ_x_ and ρ_N_ for the major uniform region of the film closely match theoretical values of ρ_x_ = 13.1 × 10^−6^ Å^−2^ and ρ_N_ = 3.17 × 10^−6^ Å^−2^, corresponding to an ideal mixing of PTB7 and PCBM in a 1:1.5 *w*/*w* ratio (equivalent to a volume ratio of 47:53). This consistency suggests a uniform distribution of PTB7/PCBM along the through-thickness direction of the film, mirroring the composition of the spin-coating solution.

**Figure 2:**
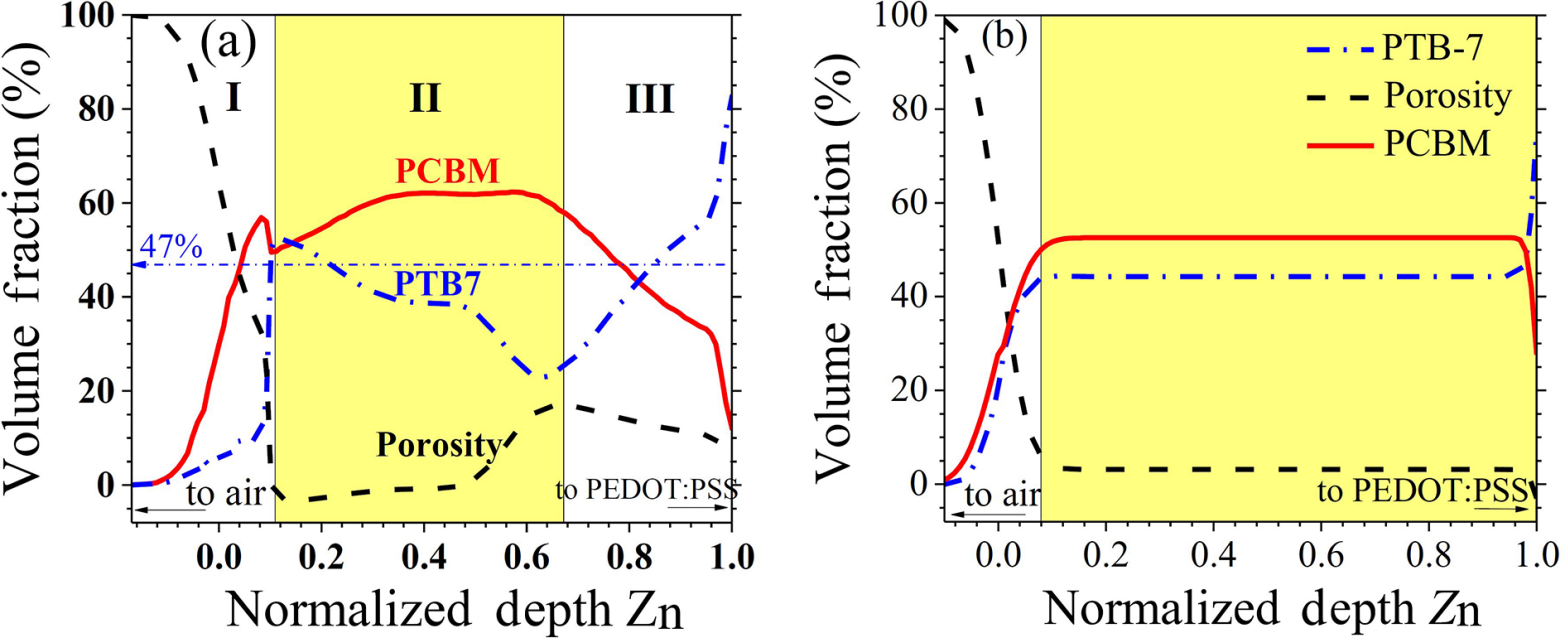
(*a*) Depth-dependent volume fraction (composition) profiles of PTB7 (blue dash–dotted curve) and PC_71_BM (red solid curve), together with porosity (black dashed curve), for the N-1.5 film along the film depth direction *Z*_n_ (140 nm film thickness). The (I) surface zone, (II) main active layer and (III) interface zone above the PEDOT-PSS conducting layer are marked. The sadh–dotted line for 47% marks a reference volume fraction of PTB7 in the spin-cast solution. (*b*) Parallel information extracted for the D-1.5 film of a film thickness of 95 nm, showing a much smoother transition zone from the active layer to the PEDOT:PSS conducting layer.

Fig. 2 ▸ showcases the through-thickness composition (*i.e.* depth-dependent volume fractions of PC_71_BM and PTB7) profiles of the N-1.5 and D-1.5 films, including the volume fraction of film porosity, with both film thicknesses normalized to unity (*Z*_n_ = 1) for comparative analysis. The volume fraction profiles are deduced using the two contrast sets of ρ_x_ and ρ_N_ profiles in Fig. 1 ▸, weighted by the respective volume fractions of the two components and the porosity contribution, as detailed in the data analysis section (Liu *et al.*, 2011 ▸; Kang *et al.*, 2018 ▸). The composition profiles for the N-1.5 film, depicted in Fig. 2 ▸(*a*), reveal three distinct structural zones within the film: (I) a porous surface zone, (II) the main active layer and (III) a transition zone prior to the PEDOT conducting layer. The surface zone constitutes about 10% of the total film thickness and is rich in PC_71_BM (55% by volume) but has less PTB7 (about 10% by volume). This layer also exhibits a high porosity volume fraction of approximately 35% within its roughly 15 nm thickness. The main active layer is notably enriched with PC_71_BM, revealing significant phase separation between PC_71_BM and PTB7, especially as the normalized film depth (*Z*_n_) exceeds 0.5, leading to increased porosity indicative of this separation. In the transition zone towards the conducting layer composed of PEDOT and polystyrene sulfonate (PSS), there is a reversal in concentrations – PCBM depletes and PTB7 enriches – accompanied by notable interfacial porosity.

The composition profiles for the D-1.5 film, as shown in Fig. 2 ▸(*b*), display significantly improved homogeneity across approximately 90% of the film’s thickness, with a marked reduction in phase segregation of the components at both the surface layer and the interface zone to the PEDOT-PSS conducting layer. The volume fractions of PTB7 and PC_71_BM in the active layer closely match the initial mixing ratios used in the spin-coating solution, with PTB7 at 47 vol% and PC_71_BM at 53 vol% (largely equivalent to the 1:1.5 *w*/*w*ratio). This homogeneity suggests that the addition of DIO effectively enhances the uniformity of the film’s composition across the film, including the surface and interfaces, thereby improving the film’s PSC device efficiency.

### 3.2. Surface structures and film porosity

**Figure 3:**
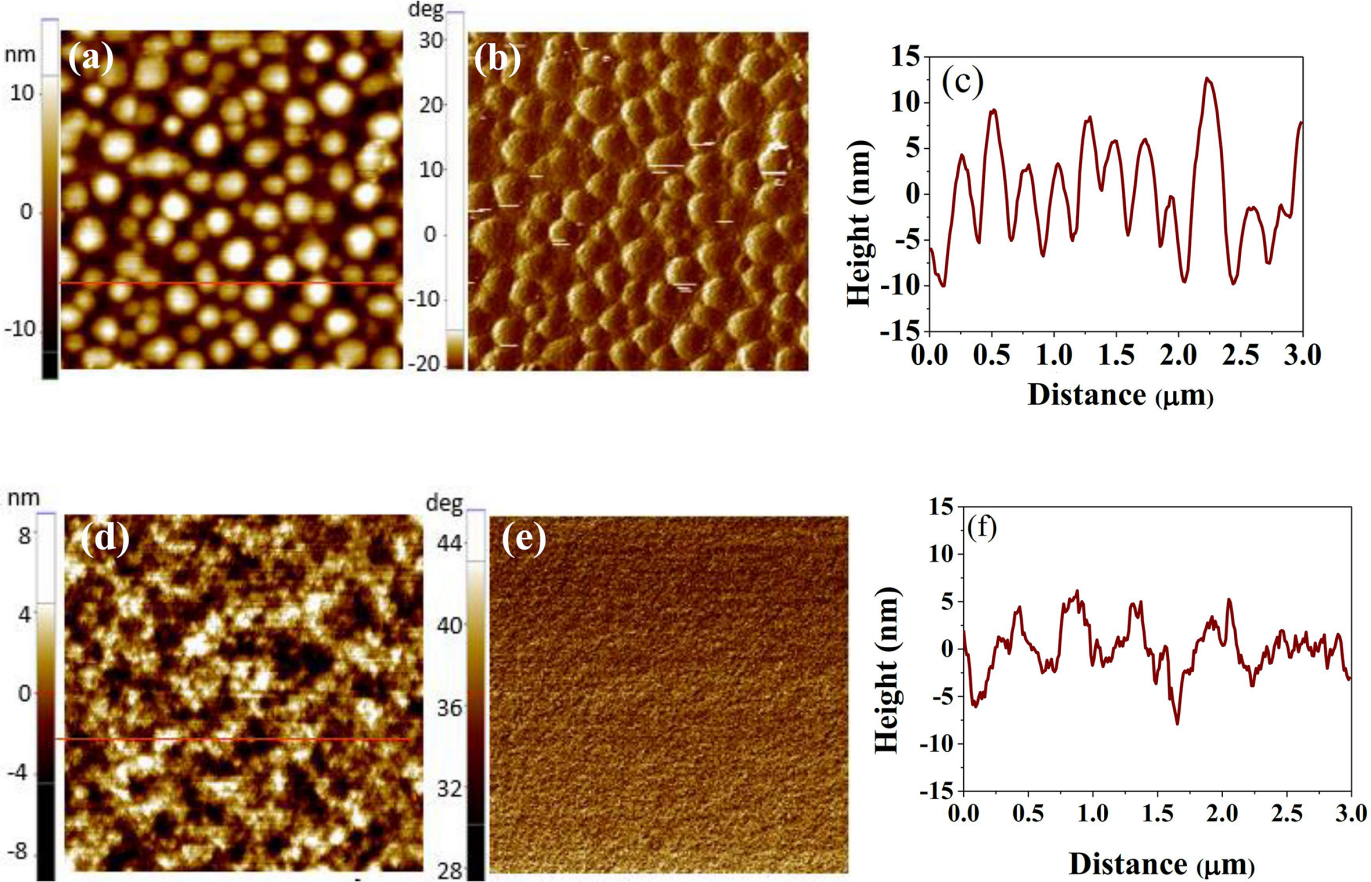
(*a*) Surface topography, (*b*) phase contrast images (3 × 3 µm) and (*c*) 1D morphological cut along the red line marked in (*a*) for the N-1.5 film. (*d*)–(*f*) are the corresponding information for the D-1.5 film.

Fig. 3 ▸(*a*) displays the AFM topography of the N-1.5 film, highlighting large, isolated spherical islands on the surface with a center-to-center spacing of 300–400 nm in the in-plane direction [Fig. 3 ▸(*c*)]. The 1D topography analysis in the vertical direction shows a significant peak-to-valley value (*R*_pv_) of approximately 23 nm, as detailed in Fig. 3 ▸(*c*), which correlates with the surface layer discussed in Fig. 2 ▸(*a*). The root-mean-square roughness (*R*_r.m.s._) measures 5.3 nm. The phase image in Fig. 3 ▸(*b*) indicates that these islands are highly rigid, suggesting they are PC_71_BM-enriched domains. Given that PC_71_BM has a significantly higher modulus (12 GPa) compared with PTB7 (1.1 GPa), these features align with the rough, PCBM-enriched surface layer described by the XR/NR results, accompanied by about 35% surface porosity (or corrugated surface). Smaller domains, which appear as dimmer images, hint at sublayer characteristics visualized through the porous surface layer. In contrast, Figs. 3 ▸(*d*)–3(*f*) for the D-1.5 film illustrate a much more uniform surface morphology, with reduced *R*_pv_ to 14 nm and *R*_r.m.s._ to 2.6 nm. These figures align with the smoother composition profiles and reduced surface roughness observed in the XR/NR results for the D-1.5 film detailed in Fig. 2 ▸(*b*), showing that the addition of DIO in film processing significantly refines the surface morphology of the film.

### 3.3. XPS for through-thickness composition

**Figure 4:**
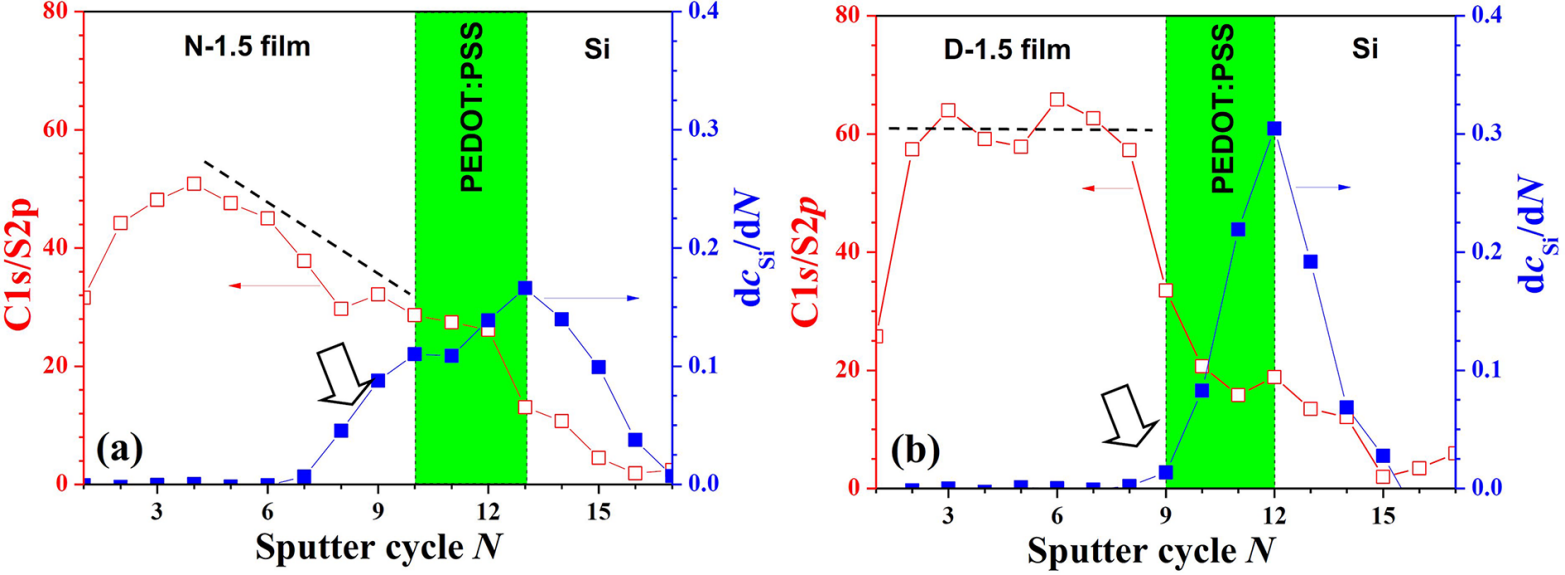
(*a*) The through-thickness C/S (or C_1*s*_/S_2*p*_) and d*c*_Si_/d*N* profiles deduced from XPS (in terms of sputter cycle *N*) for N-1.5, and (*b*) the corresponding data for the D-1.5 films. The d*c*_Si_/d*N* peak position corresponds to the interface between the PEDOT:PSS layer and the Si substrate. The big arrow in (*a*) marks the porosity effect on the non-trivial d*c*_Si_/d*N* values observed before reaching the PEDOT:PSS layer in the N-1.5 film.

Depth-resolved XPS measurements were conducted to confirm the through-thickness composition profiles for both the N-1.5 and D-1.5 films. In the XPS spectra shown in Fig. S3, the sulfur composition peak is attributed to the thio­phene rings in PTB7, while the carbon absorption peak results from contributions of both PTB7 and PC_71_BM. The C/S ratio, defined as the carbon intensity divided by the sulfur intensity [shown in Fig. 4 ▸(*a*)], generally highlights a PCBM-enriched upper layer in the N-1.5 film and a more uniformly distributed PC_71_BM–PTB7 in the D-1.5 film, consistent with previous XR/NR findings [*cf*. Figs. 2 ▸(*a*), 2 ▸(*b*)]. Further analysis of film porosity was carried out using depth-dependent Si signals from the XPS data (Fig. S3). The intensity changes of Si-2*p* signals (d*c*_Si_/d*N*) along the film depth, where *c*_Si_ represents the measured intensity of Si-2*p* at each sputtering depth and *N* denotes the number of sputtering cycles, were calculated. This analysis showed an earlier and steeper rise in the d*c*_Si_/d*N* profile for the N-1.5 film compared with the D-1.5 film [Figs. 4 ▸(*a*), 4 ▸(*b*)], indicating greater porosity or transparency to the Si substrate in the N-1.5 film. These results align well with the porosity and composition distributions previously deduced from contrast XR/NR analyses (Fig. 2 ▸), confirming the distinct structural differences of these two films.

### 3.4. Aggregation and crystalline nanodomains

**Figure 5:**
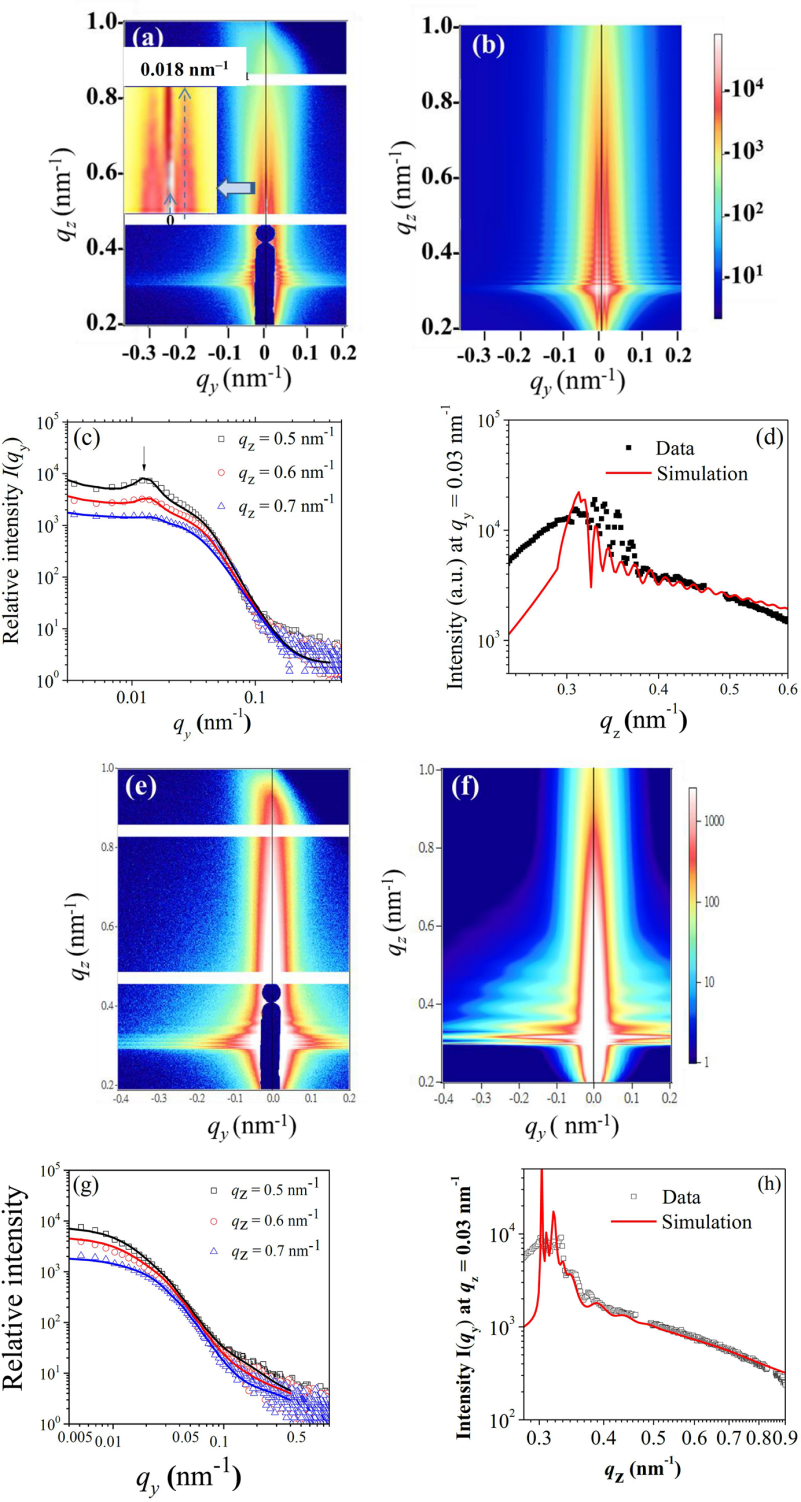
(*a*) Observed and (*b*) simulated 2D GISAXS patterns of the N-1.5 film. Inset in (*a*) is a zoomed-in view of the scattering stripes centered at *q*_*y*_ ≃ 0.018 nm^−1^. Selected comparisons of the measured (symbols) and simulated (solid curves) GISAXS line profiles along (*c*) in-plane and (*d*) out-of-plane directions of the corresponding 2D GISAXS patterns, at the respective *q*_*z*_ or *q*_*y*_ positions indicated. (*e*) Observed and (*f*) simulated 2D GISAXS patterns for the D-1.5 film; measured and fitted GISAXS line profiles in the (*g*) in-plane and (*h*) out-of-plane directions.

The segregation characteristics of PTB7 and PC_71_BM in the through-thickness direction of the film are linked to local phase separation in the in-plane direction, through GISAXS analysis. As depicted in Fig. 5 ▸(*a*), the 2D GISAXS pattern of the N-1.5 sample shows strong vertical stripes at a *q*_*y*_ value (the scattering vector component along the film in-plane direction) of approximately 0.00180 Å^−1^. This pattern indicates that the phase-separated domains are not only highly oriented but also ordered along the in-plane direction (Renaud *et al.*, 2009 ▸). These nanodomains have an average spacing of about 350 nm, calculated using Bragg’s law from the 2π/*q*_*y*_ value. GISAXS measurements at two additional incident angles, 0.12° and 0.2°, provide insights into the structural variations along the depth direction of the PTB7–PC_71_BM films. The 0.12° incidence, being below the film’s critical angle for total reflection [which is approximately 0.13° at 10 keV, as estimated using the fitted ρ_x_ value of 13.5 × 10^−6^ Å­^−2^ depicted in Fig. 1 ▸(*b*)], enhances scattering from the film’s surface features. This resulted in a peaked position shifted to a higher *q*_*y*_ value of 0.0021 Å^−1^, suggesting a smaller mean spacing of approximately 285 nm for PC_71_BM nanodomains segregated to the near-surface region. In contrast, the 0.2° angle incidence, which allows for greater penetration, reveals an even smaller *q*_*y*_ ≃ 0.00165 Å^−1^ for 380 nm *d* spacing in the deeper film. These results show a trend of successively increased mean spacing between PC_71_BM-enriched nanodomains, suggesting a decreasing PC_71_BM concentration deeper within the film (Fig. S4). This pattern aligns with the decreasing ρ_x_ values along the film depth, indicative of a gradient in PC_71_BM concentration across the film’s thickness, as consistently illustrated in Fig. 2 ▸(*a*).

We have constructed a model structure for the N-1.5 film on the basis of the structural parameters used in the three-sublayer model of ρ_x_ obtained from XR data analysis [Fig. 1 ▸(*b*)] to fit the GISAXS data. Assuming that the GISAXS scattering features are dominated by PC_71_BM-enriched oblate aggregates distributed in a PTB7 matrix, we optimized the sublayer thicknesses and the size, shape and distribution of the oblates embedded within each of three sublayers using the *fitGISAXS* software package (Babonneau, 2010 ▸). As a result, the simulated 2D GISAXS pattern could capture the major features observed for the N-1.5 film as shown in Figs. 5 ▸(*a*) and 5 ▸(*b*) using the fitted structural parameters summarized in Table 1 ▸. The fitted three-layer model features a surface layer of 13 nm thickness embedded with oblates of major axis 2*a* = 11 nm and minor axis 2*b* = 270 nm, having a paracrystal ordering *d* spacing of 285 nm along the in-plane direction. Further, these large surface oblate nanodomains are oriented with their major axis parallel to the surface normal, highlighting highly anisotropic structural features [complementary to the AFM image in Fig. 3 ▸(*a*)]. Beneath this are two major sublayers of 63 and 69 nm thickness, respectively, embedded with large oblates (2*a* = 20 nm, 2*b* = 260 nm, and a paracrystal *d* spacing = 350 nm) and randomly distributed small oblates (2*a* = 4 nm and 2*b* = 80 nm).

Comparisons between the line-cut GISAXS profiles in the in-plane *q*_*y*_ direction from the simulations and the GISAXS measurements [Fig. 5 ▸(*c*)] show good alignment, supporting our finding that the simulation adequately represents the in-plane structural characteristics of the film. However, some deviations are observed in the line-cut GISAXS profile along the *q*_*z*_ direction [Fig. 5 ▸(*d*)] from the 2D GISAXS pattern. This suggests that, while the three-layer model effectively captures in-plane features, it may not fully account for out-of-plane structural variations. This discrepancy illustrates the complexities in modeling the scattering features observed in the 2D GISAXS pattern and suggests that further refinement of the structural model, potentially incorporating additional sublayers, may be necessary to achieve a more comprehensive understanding of the film’s architecture.

The GISAXS analysis of the D-1.5 film shows a notable absence of the N-1.5 scattering stripes in the very low *q* region [Fig. 5 ▸(*e*)] from phase segregation, suggesting a more homogeneous distribution of PC_71_BM-enriched nanodomains compared with the N-1.5 film. Additionally, the scattering intensity for the D-1.5 film extends further into the higher-*q* region, indicating the presence of smaller and more densely packed scattering nanodomains that decay more slowly in intensity as *q* values increase. To fit the 2D GISAXS pattern [Fig. 5 ▸(*f*)], a one-layer model of ρ_x_, derived from XR results [Fig. 1 ▸(*d*)], was employed. This model included disperse spheres with 5 nm diameter following a 40% Schultz size distribution, together with part of the spheres forming fractal aggregates. The fractal aggregates within the model are described with a fractal dimension of *D* = 2.0 and a correlation length of ξ = 40 nm. The characteristic size of the fractal structure approximated by twice the radius of gyration 2*R*_g_ = 4ξ[*D*(*D* + 1)]^1/2^ (Teixeira, 1988 ▸; Jhuo *et al.*, 2016 ▸) is about 390 nm, which aligns roughly with the fractal-like structures observed in AFM imaging [Figs. 3 ▸(*d*), 3 ▸(*f*)]. The sophisticated modeling underscores the significant impact of DIO in refining PC_71_BM dispersion in the PTB7 matrix, from large and discrete PC_71_BM oblate domains in the N-1.5 film to the fractal aggregates comprising small PC_71_BM nanodomains in the D-1.5 film. We note that a model-independent parameter like correlation length was proposed in a previous report (Ehmann *et al.*, 2015 ▸) to describe qualitatively structural changes revealed from line-cut GISAXS profiles in real-space units.

GIWAXS analysis performed on both N-1.5 and D-1.5 films reveals corresponding differences in their structural properties. Notably, the D-1.5 film shows a suppressed aggregation hump of PCBM at approximately *q* ≃ 1.4 Å^−1^, in contrast to the more pronounced aggregation observed in the N-1.5 film (Fig. S5). This consistently suggests that the presence of the additive DIO significantly reduces the aggregation behavior of PC_71_BM in the D-1.5 film, leading to the significantly reduced aggregation peak. Furthermore, the GIWAXS data shown in Fig. S5 reveal minor influences on the rather weak (100) packing of PTB7 in the D-1.5 film compared with the N-1.5 film (Jhuo *et al.*, 2016 ▸). This observation implies that the additive mainly suppresses the phase segregation of PC_71_BM.

**Figure 6:**
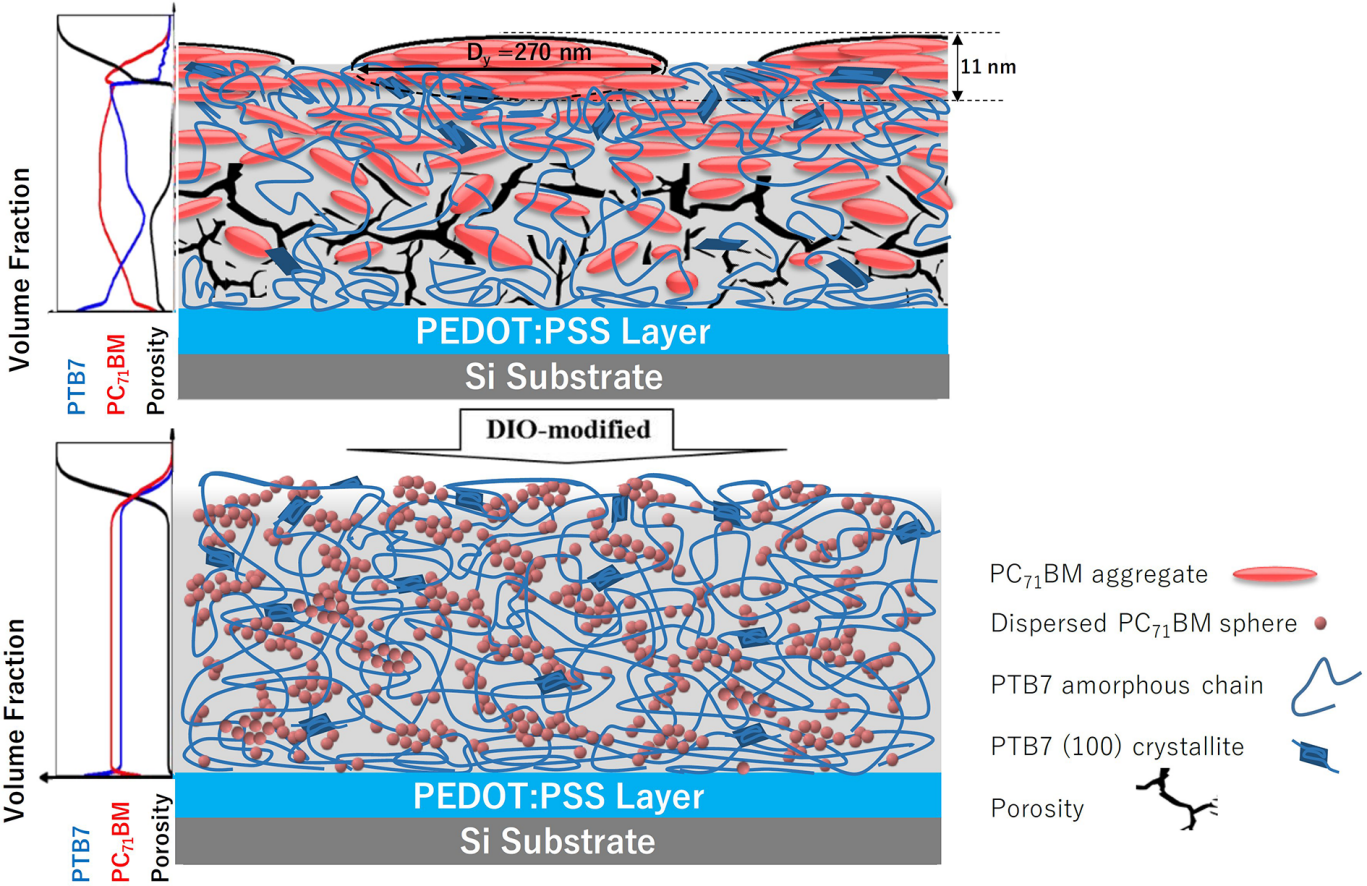
Cartoons illustrate the nanostructural features of PC_71_BM nanodomains for the N-1.5 (top) and D-1.5 (bottom) films, including the through-thickness composition profiles illustrated on the left-hand side. Note that the PC_71_BM aggregates (in red) are enriched and enlarged at and near the surface of the N-1.5 film. In contrast, relatively small PC_71_BM aggregates are distributed homogeneously in the D-1.5 film, with porosity largely eliminated.

Fig. 6 ▸ encapsulates the structural distinctions observed in the N-1.5 and D-1.5 films. The N-1.5 film, processed without the additive DIO, displays pronounced segregation, with PC_71_BM concentrating predominantly in the upper section of the film. This segregation leads to the formation of large, ordered PCBM-rich oblate nanodomains near the film surface, while the bottom zone becomes enriched with PTB7 and shows significant porosity [as depicted in Fig. 6 ▸(*a*)]. Conversely, in the D-1.5 film, the addition of DIO in the film processing substantially reduces the aggregation of PC_71_BM into relatively dispersed small aggregates with fractal-like interconnections within the PTB7 film matrix [Fig. 6 ▸(*b*)].

### 3.5. Binding energy and phase segregation

**Figure 7:**
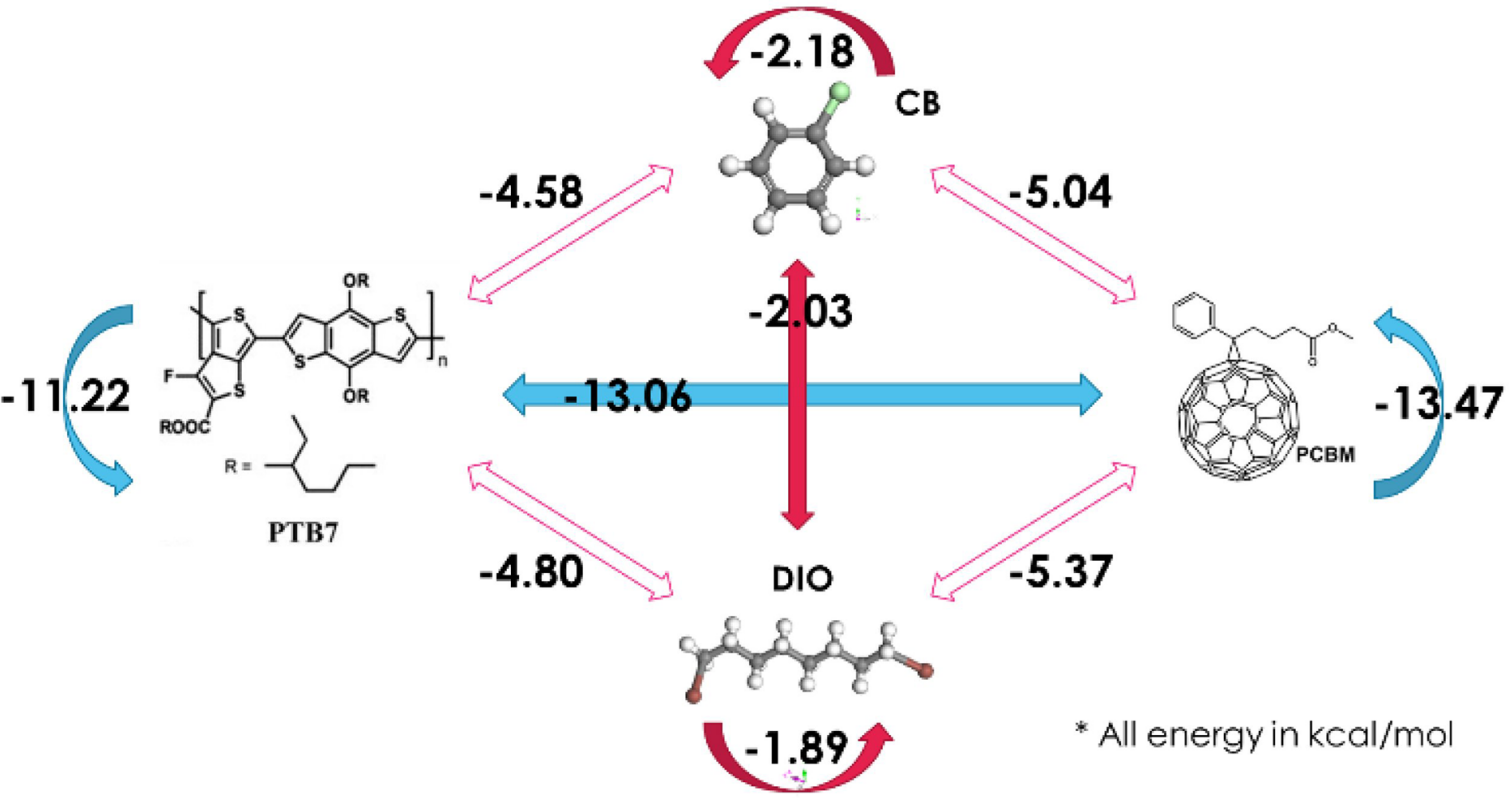
Cartoon of the pair binding energy (as indicated; in units of kcal mol^−1^) of the four components in a CB solution containing DIO, PC_71_BM and PTB7 for spin-coating.

To better understand how DIO influences phase separation and the resulting film morphology, we conducted energy pair interaction calculations among the four components: PC_71_BM, PTB7, DIO and CB. These were performed using Monte Carlo simulations and DFT calculations (*Dmol3*) within the *Materials Studio* software package. Results from these detailed calculations are summarized in Table S2 and Fig. 7 ▸. They show that PC_71_BM has the highest self-affinity with a binding energy of −13.47 kcal mol^−1^. PTB7 also demonstrates a strong affinity towards PC_71_BM, with a binding energy of −13.06 kcal mol^−1^, which is notably higher than PTB7’s self-affinity of −11.22 kcal mol^−1^. This suggests that the presence of PC_71_BM would weaken the crystallization of PTB7 within the active layer. Additionally, DIO shows a substantially better affinity for PC_71_BM (−5.37 kcal mol^−1^) compared with PTB7 (−4.08 kcal mol^−1^).

The significantly lower affinities of the solvent CB and the additive DIO to PC_71_BM and PTB7, compared with the self-affinities of the latter, indicate low solute solubility, and phase segregation would be sensitive to the changes of the solute concentrations over the solubility limits during a spin-coating process with solvent evaporation. DIO, which has a higher boiling point (b.p. = 332.5 °C) compared with the more volatile CB (b.p. = 132 °C), likely influences the solvent-evaporation kinetics during film spin-casting, leading to the modulated phase segregation and aggregation of PC_71_BM observed in the spin-cast of the D-1.5 film.

### 3.6. Phase segregation kinetics during spin-casting

**Figure 8:**
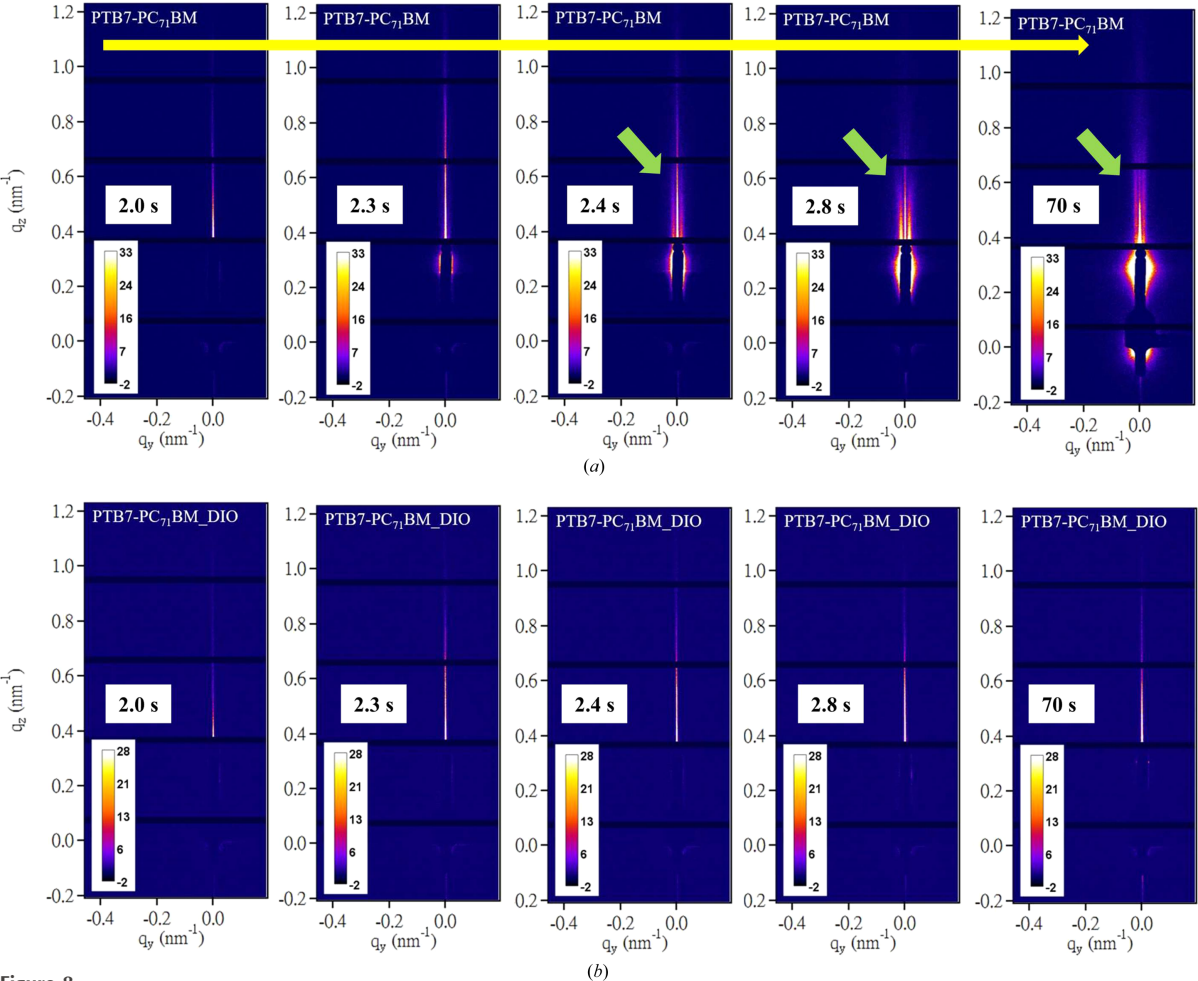
(*a*) Representative time-dependent GISAXS patterns taken with 100 ms resolution during spin-coating of the PTB7–PC_71_BM film from the sample solutions of CB without additive, over the spin-coating time indicated. The vertical arrows in (*a*) indicate the emergence and development of the vertical scattering stripes at *q*_*y*_ ≃ 0.002 Å^−1^ over *t* = 2.4–2.8 s of the spin-coating time. (*b*) Parallel GISAXS patterns for the case with 3% DIO, showing no scattering stripes (see the movies in the supporting information for the complete spin-coating process).

**Figure 9:**
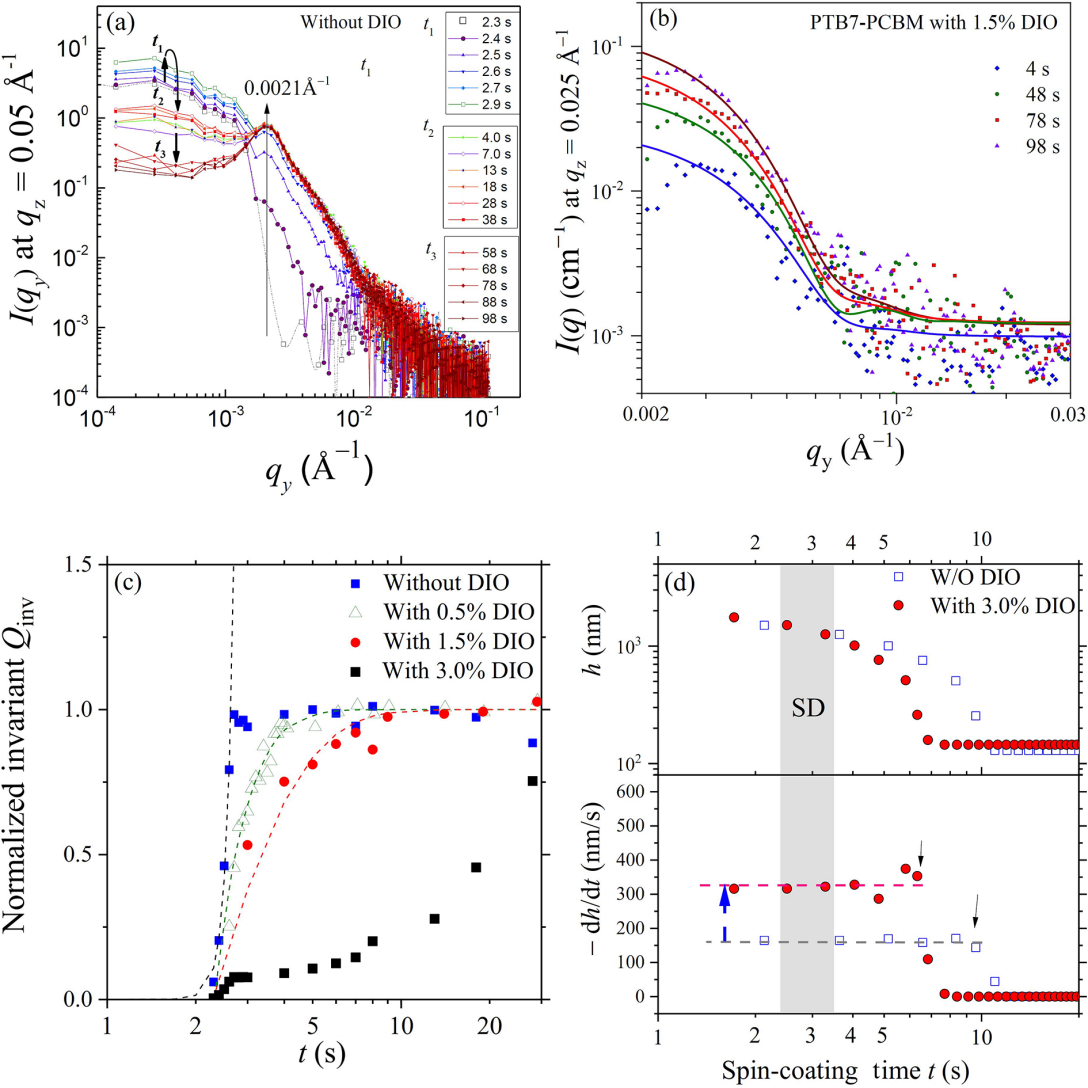
(*a*) Time-resolved, in-plane GISAXS profiles selectively extracted at *q*_*z*_ = 0.05 Å^−1^ from the corresponding 2D GISAXS patterns in Fig. 8 ▸(*a*), measured during spin-coating of the CB solution of PTB7/PC_71_BT, without DIO. The thick and curved arrows indicate the growth and decay of the intensity in the very low *q* region (<0.001 Å^−1^) during the early (*t*_1_), intermediate (*t*_1_–*t*_2_) and late (*t* > *t*_3_) spin-coating stages. The thin long arrow indicates the growth and saturation of the SD peak at *q*_*y*_ ≃ 0.002 Å^−1^. (*b*) Selected in-plane GISAXS profiles extracted at *q*_*z*_ = 0.025 Å^−1^ (see Fig. S6 for details) for the film processed with 1.5% DIO. Data at *t* = 4, 48, 78 and 98 s are selectively fitted (solid curves) using a sphere model of radii of 53 ± 8, 56 ± 4, 60 ± 4 and 58 ± 3 nm, respectively. (*c*) The growth behaviors of *Q*_inv_ (extracted from the corresponding time-resolved in-plane GISAXS profiles) during spin-coating of the PTB7–PC_71_BM films from the CB solutions, with the DIO concentrations indicated. (*d*) Time-dependent film thickness *h* (top) and film thinning rate d*h*/d*t* (bottom) measured using UV–Vis reflectance during film spin-coating without and with 3% DIO. The horizontal dotted lines label the constant-evaporation regions. The shaded zone marks the timing of SD in (*a*).

To investigate the effects of DIO on phase segregation during the formation of PTB7–PC_71_BM films of N-1.5 and D-1.5, we conducted time-resolved GISAXS–GIWAXS measurements with 100 ms resolution during spin-coating (see the videos in the supporting information) of the films. For the cases without DIO, the GISAXS patterns [Fig. 8 ▸(*a*)] captured 2.4 s after initiation of the spin-coating process showed the emergence and fast development of vertical scattering stripes at approximately *q*_*y*_ = 0.002 Å^−1^. These patterns revealed that the LLPS of the solution film proceeded via the spinodal decomposition (SD) mechanism of polymer blends (Vaynzof *et al.*, 2011 ▸; Heriot & Jones, 2005 ▸; Toolan *et al.*, 2013 ▸). This mechanism is characterized by rapid demixing from a homogeneous phase into bi-continuous phases, driven by concentration fluctuations that allow for spontaneous phase segregation as opposed to localized nucleation and growth (van Franeker *et al.*, 2015*c* ▸). The consistency of the peak position over time indicated that the composition fluctuations converged rapidly to a spinodal wavelength (λ_s_), with a mean spacing corresponding to the SD peak observed at *q*_s_ = 0.002 Å^−1^ (Chuang *et al.*, 2007 ▸; Cahn & Hilliard, 1958 ▸; Cahn & Hilliard, 1959 ▸). The corresponding *d* spacing of 314 nm deduced from the SD peak position (2π/*q*_s_) aligns closely with the mean spacing of PC_71_BM nanodomains previously revealed from AFM [Fig. 3 ▸(*c*)] and static GISAXS (Table 1 ▸). These observations suggest that the final film morphology is largely determined in the early stage of spin-coating. Interestingly, we found that the addition of 3% DIO to the spin-coating solution completely suppressed this fast SD type of phase segregation, as shown in Fig. 8 ▸(*b*), with no vertical scattering stripes. Nevertheless, Fig. 9 ▸(*c*) indicates that phase separation could still proceed except with much slower kinetics [Fig. 9 ▸(*b*)], leading to nanostructural features differing from those of the N-1.5 film, as illustrated in Fig. 6 ▸.

In our previous study (Wu *et al.*, 2017 ▸), three stages of the spin-coating process with CB solutions of P3HT/PC_61_BM could be observed: stage I, the flow-off stage of quickly reduced film thickness; stage II, constant-evaporation stage for increase of solute concentration; and stage III, the late stage with slowing down of film thinning due to slow depletion of residual solvent deep inside the film. The three stages are mainly characterized by the features of the thinning rate d*h*/d*t* of the film thickness *h* observed over the spin-casting process, using an *in situ* UV–Vis interferometer (Wu *et al.*, 2017 ▸; Heriot & Jones, 2005 ▸; Haas *et al.*, 2000 ▸). For spin-casting with the CB solutions containing PTB7 and PC_71_BM for the N-1.5 and D-1.5 films, Fig. 9 ▸(*d*) shows the time-dependent *h* and d*h*/d*t* of the solution films, exhibiting features of the constant-evaporation stage II and stage III. We note that the flow-off stage I could not be observed in either case.

The time-resolved in-plane GISAXS profiles selectively extracted at *q*_*z*_ = 0.05 Å^−1^ for the prominent phase segregation peak of the N-1.5 film further characterize the features of the LLPS process. Combined with the observed film thinning behavior, these GISAXS profiles [Fig. 9 ▸(*a*)] show how the film’s major phase segregation could be largely complete within 1 s at the beginning of the constant-evaporation stage [Fig. 9 ▸(*d*)]. During this stage, the intensity in the very low *q* region (<0.001 Å^−1^) increases along with the SD ordering peak intensity at about *q*_s_ ≃ 0.002 Å^−1^ and quickly saturates within 1 s (at the spin-coating time *t* = 2–3 s). This suggests that concentration fluctuations amplify quickly at a fixed wavelength (or *q*_s_), leading to an increase of the SD peak intensity as phase segregation begins to set in. We note that this spinodal peak position *q*_s_ [Fig. 9 ▸(*a*)] is consistent with the interference peak observed in the final spin-cast film (Fig. 5 ▸) for the large surface PC_71_BM aggregates [Fig. 6 ▸(*a*)], suggesting that PC_71_BM phase segregation (via a SD process) from the homogeneous mixture was initiated from the solution film surface shortly after the start of the spin-coating process. The UV–Vis result indicates that the corresponding solution film thickness is *ca* 1 µm, when the spin-casting process evolves into the constant-evaporation stage (Li *et al.*, 2014 ▸; Chambon & Winter, 1987 ▸), with a constant film thinning rate of d*h*/d*t* = −150 nm s^−1^ [Fig. 9 ▸(*d*)]. As also shown in a previous report (Wu *et al.*, 2017 ▸), the solute concentration of the spin-coating solution film would start to increase over the miscibility limit of the mixture in this constant-evaporation stage, thereby triggering the surface-initiated phase separation. During the course of stage II (∼10 s), the very low *q* intensity starts to decrease with the sharpening of the SD peak (of stable peak intensity), which is attributed to refinement of the packing order of the phase-separated PCBM-rich domains, leading to the interference suppression on the form factor scattering in the low-*q* region [Fig. 9 ▸(*a*)]. In the late stage III (*t >* 10 s), the scattering profiles are largely overlapped, reflecting the fact that phase segregation of the film is largely completed in stage II of the constant-evaporation regime, in the length scale (*q* range) monitored, as quantitatively revealed by the scattering invariant *Q*_inv_ shown in Fig. 9 ▸(*c*). We note that *Q*_inv_ is extracted from the in-plane, line-cut GISAXS profiles, associated with the changes of the system’s heterogeneity along the in-plane direction. However, the results are consistent with the picture obtained from our previous 3D modeling of the 2D GISAXS patterns.

We note that the simultaneously measured GIWAXS during the *in situ* spin-coating of the PTB7–PC_71_BM blend film shows no observable changes in GIWAXS profiles or crystalline peak formation during the fast development of GIWAXS patterns (see the movie files in the supporting information). This may be attributed to the weak crystallization (lower self-affinity) of PTB7 in the composite film as revealed from Fig. 7 ▸. Therefore, the SD is attributed largely to the segregation of PC_71_BM from the mixture for self-aggregation due to its higher self-binding energy (*cf*. Fig. 7 ▸).

### 3.7. DIO effects

**Figure 10:**
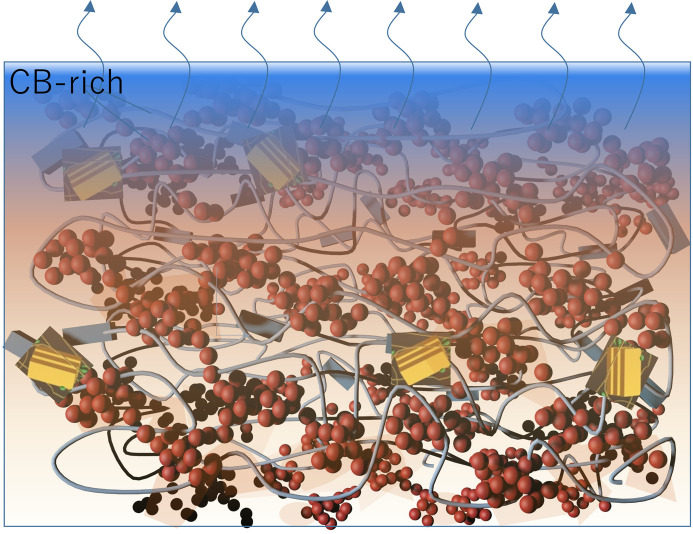
Schematic illustration of the effects of the DIO additive on modulating phase segregation in the PTB7–PC_71_BM blend during spin-coating from a CB/DIO (97:3 *v*/*v*) solution. The high boiling point of DIO accelerates the evaporation of the low-boiling-point CB during the spin-coating process. In the near-surface region (depicted by the graduated blue zone), the rapid evaporation of CB with high flux prevents excessive solute concentration at the surface, thereby inhibiting rapid phase segregation via spinodal decomposition. As CB quickly evaporates, the remaining PC_71_BM–PTB7 mixture becomes kinetically trapped in the DIO-rich environment, where the slow segregation of PC_71_BM leads to the formation of small PC_71_BM aggregates (represented by red spheres) a few nanometres in size. These small aggregates further interconnect into fractal-like clusters within the PTB7/DIO matrix, which consists of both amorphous chains and crystalline regions (depicted as wires and blocks). DIO, accounting for approximately 24% of the film’s volume fraction (as estimated from the solution composition after the exhausted evaporation of CB), is represented by the irregular orange patches. In contrast, Fig. 6 ▸ illustrates a film from which DIO was removed by vacuum evacuation.

According to the complementary time-resolved GISAXS and UV–Vis reflectance results, the SD phase separation for the N-1.5 film occurs and reaches saturation mainly at the beginning of the spin-casting time, *t* = 2–3 s, in the constant-evaporation regime, when the solution film thickness is about 1.5 µm. During the remainder of the spin-coating process, the film thickness reduces continuously to about 200 nm with not much phase segregation. With 3% DIO added to the spin-coating solution, Fig. 9 ▸(*d*) indicates that the d*h*/d*t* rate is increased drastically to 320 nm s^−1^ from 160 nm s^−1^ in the case without DIO. Consequently, the constant-evaporation stage with DIO is shortened to 6 s, compared with 12 s for the N-1.5 film spin-coating [Fig. 9 ▸(*d*)]. For comparison, the evaporation rate measured for pure CB under similar conditions is *ca* 550 nm s^−1^. These results illustrate that DIO of a high boiling point 333 °C, compared with 132 °C of CB, plays a crucial role in the spin-coating process of PTB7–PC_71_BM films. This DIO-accelerated CB evaporation presumably provokes a fast CB flow from a deeper zone to the surface of the solution film. Consequently, the high-flux CB may occupy significantly the near-surface zone, as illustrated in Fig. 10 ▸, thereby preventing solute concentrating at the surface for SD during the shortened constant-evaporation stage (Wu *et al.*, 2017 ▸; Heriot & Jones, 2005 ▸; Toolan *et al.*, 2013 ▸). After the major solvent CB is quickly depleted, the mixture is kinetically trapped in the solidifying film with reduced mobility, resulting in a much slower phase segregation process.

To further elucidate the DIO effects, we also conducted additional GISAXS measurements of *in situ* spin-casting of sample solutions with added 0.5% and 1.5%(*v*/*v*) DIO. The results (see Fig. S6) indicate a progressive slowing down of SD-type phase segregation with increasing DIO concentration, as quantitatively illustrated by the successive slowdown in growth behavior of *Q*_inv_ shown in Fig. 9 ▸(*c*). These results reveal that the fast SD type of phase separation is systematically delayed and altered by the successively increased DIO concentration. We note that the phase separation kinetics for the case with 3% DIO were very slow and the corresponding time-resolved GISAXS profiles measured over the whole spin-coating process are of low intensity. Therefore, we analyzed the time-resolved GISAXS profiles for the case with 1.5% DIO with better intensity, which also exhibit no observable SD type of phase separation (see Fig. S6). These GISAXS profiles [Fig. 9 ▸(*b*)] can be fitted using a sphere model (for simplicity) of similar radii near 55 ± 5 nm; this size scale corresponds to the PC_71_BM-rich clusters illustrated in Fig. 6 ▸ (or the correlation length in Table 1 ▸ for the D-1.5 film). These results suggest that the phase segregation mechanism during spin-coating of the PTB7–PC_71_BM mixture can be modulated from the SD type without DIO to the nucleation-driven process when the DIO concentration is roughly above 1.5%. These results support the empirical strategy of using 3% DIO in processing PSC device films for better control of film morphology and optimal PCE (Kim *et al.*, 2015 ▸).

## 4. Conclusions

We have delineated the control mechanisms and kinetics of phase segregation during the spin-coating of PTB7–PC_71_BM, a widely studied PSC. From our detailed structural characterization, including surface morphology, composition profiles across the film’s thickness, and static features and kinetics of the nanostructure formation, we have concluded that the morphology is influenced by a rapid liquid–liquid phase segregation of the donor and acceptor components via a spinodal decomposition process during the beginning of the constant-evaporation stage of spin-coating. Importantly, we have elucidated that this rapid phase segregation can be altered by DIO additive into nucleation-driven slow phase segregation in the spin-coating solution. The role of additives in controlling the nanostructural evolution of spin-cast films by altering the kinetics of solvent evaporation provides mechanistic insights into phase separation during the spin-coating process. The critical insights may be of use in processing broader solution spin-cast films, including techniques like spray- or slot-die coating for large-area PSCs. Optimizing solvent evaporation control during spin-coating, either through the use of additives or by employing methods like solvent washing or solvent annealing during spin-coating, may be developed to better minimize inhomogeneities at or near the surface of functional thin films for improved performance.

## Supplementary Material

Static GISAXS/GIWAXS and in situ GISAXS data, XPS data and Monte Carlo (MC) calculation, and XR/NR data for single component. DOI: 10.1107/S1600576724010082/jl5094sup1.pdf

PTB7–PCBM with 3% DIO spin-coating. DOI: 10.1107/S1600576724010082/jl5094sup2.mp4

PTB7–PCBM without DIO spin-coating. DOI: 10.1107/S1600576724010082/jl5094sup3.mp4

## Figures and Tables

**Table:** The fitting parameters used are the X-ray SLD ρ_x_, thickness *t* and interfacial roughness σ of each sublayer, together with the major and minor axes 2*a* and 2*b* of the oblates embedded inside each sublayer with or without a paracrystal-like ordering *d* spacing (along the in-plane direction). In the lower part of the table for the one-layer model of the D-1.5 film, 2*r* is the diameter of the primary spheres of polydispersity *p*, and *D* and ξ are, respectively, the fractal dimension and the correlation length of the fractal model.

N-1.5 film	ρ_x_ (10^−6^ Å^−2^)	*t* (nm)	σ (nm)	2*a* (nm)	2*b* (nm)	*d* (nm)
Surface layer	10.0	13	7	11	270	285
Upper layer	13.5	63	0.5	20	280	350
Lower layer	11.3	69	7	4	60	–
PEDOT:PSS	12.1	40	3	–	–	–
Si substrate	20.0	–	0.5	–	–	–

**Table:** 

D-1.5 film	ρ_x_ (10^−6^ Å^−2^)	*t* (nm)	σ (nm)	2*r* (nm)	*p* (%)	*D*	ξ (nm^−1^)
Main layer	12.7	97	4	5	40	2.0	40
PEDOT:PSS	12.1	45	1	–	–	–	–
Si substrate	20.0	–	0.5	–	–	–	–
