# Supplementary material for: Modulating phase segregation during spin-casting of fullerene-based polymer solar-cell thin films upon minor addition of a high-boiling co-solvent
Source: J Appl Crystallogr. 2024 Nov 17;57(Pt 6):1871–83. doi: 10.1107/S1600576724010082 (PMC11611283; doi:10.1107/S1600576724010082)
Supplement: Supplementary file 1 [file j-57-01871-sup1.pdf]

JOURNAL OF APPLIED CRYSTALLOGRAPHY

Volume 57 (2024)

Supporting information for article:

Modulating phase segregation during spin-casting of fullerene-based polymer solar-cell thin films upon minor addition of a high-boiling co-solvent

Kuan-Hsun Lu, Wei-Ru Wu, Chun-Jen Su, Po-Wei Yang, Norifumi L. Yamada, Hong-Jun Zhuo, Show-An Chen, Wei-Tsung Chuang, Yi-Kang Lan, An-Chung Su and U-Ser Jeng

S1. . GISAXS setup

With the sample surface defined in the *xy*-plane and the X-ray (wavelength λ) beam incidence in the *xz*-plane, the scattering wavevector transfer $\boldsymbol{q}$ = (q_x, q_y, q_z) was defined by: $q_x = 2\pi\lambda^{-1}(\cos\beta\cos\phi - \cos\alpha)$, $q_y = 2\pi\lambda^{-1}(\cos\beta\sin\phi)$, and $q_z = 2\pi\lambda^{-1}(\sin\alpha + \sin\beta)$ where α and β represent the incident and exit angles and ϕ measures the scattering angle away from the *x*-*z* plane, as shown in Figure S1. The scattering wavevector *q* of the GISAXS or GIWAXS was calibrated using silver behenate, sodalite, and silicon powders. With a spin-coating speed of 1100 rpm and a sample substrate of 14×14 mm^2. A Pilatus 1M-F area detector and a CMOS flat panel detector C9728-DK, were located at sample-to-detector distances of 5092 and 160 mm, for GISAXS and GIWAXS, respectively. With an X-ray beam of 10 keV, GISAXS/GIWAXS patterns were simultaneously collected at static or with a time resolution of 100 ms – 1s at 30 °C during spin-coating. The spin-coating chamber was sealed with controlled air-flow during the whole spin-coating process.

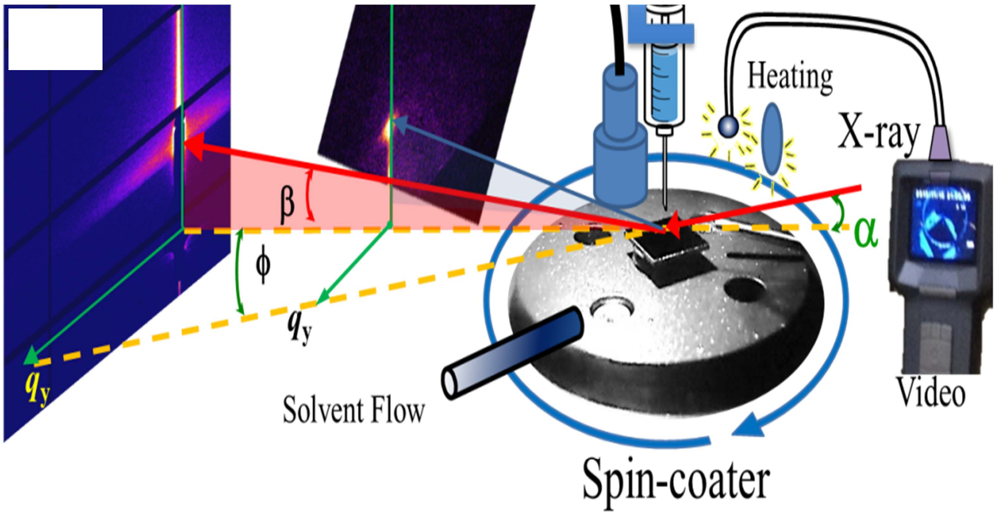

Figure S1 Schematic view of the synchronized GISAXS/GIWAXS setup, including the injector, spin-coater, an air flow system for solvent vapor evacuation, and a video system. The X-ray beam incident and scattering angles are α, β, and ϕ, as indicated. Also illustrated is the UV-vis spectrometer with normal incidence and reflection from the sample surface. The setup is reproduced from Figure 1a of the reference: Wu, W.-R., Su, C.-J., Chuang, W.-T., Huang, Y.-C., Yang, P.-W., Lin, P.-C., & Jeng, U. (2017). Adv. Energy Mater. 7, 1601842(11).

S2. X-ray and Neutron reflectivity for single component

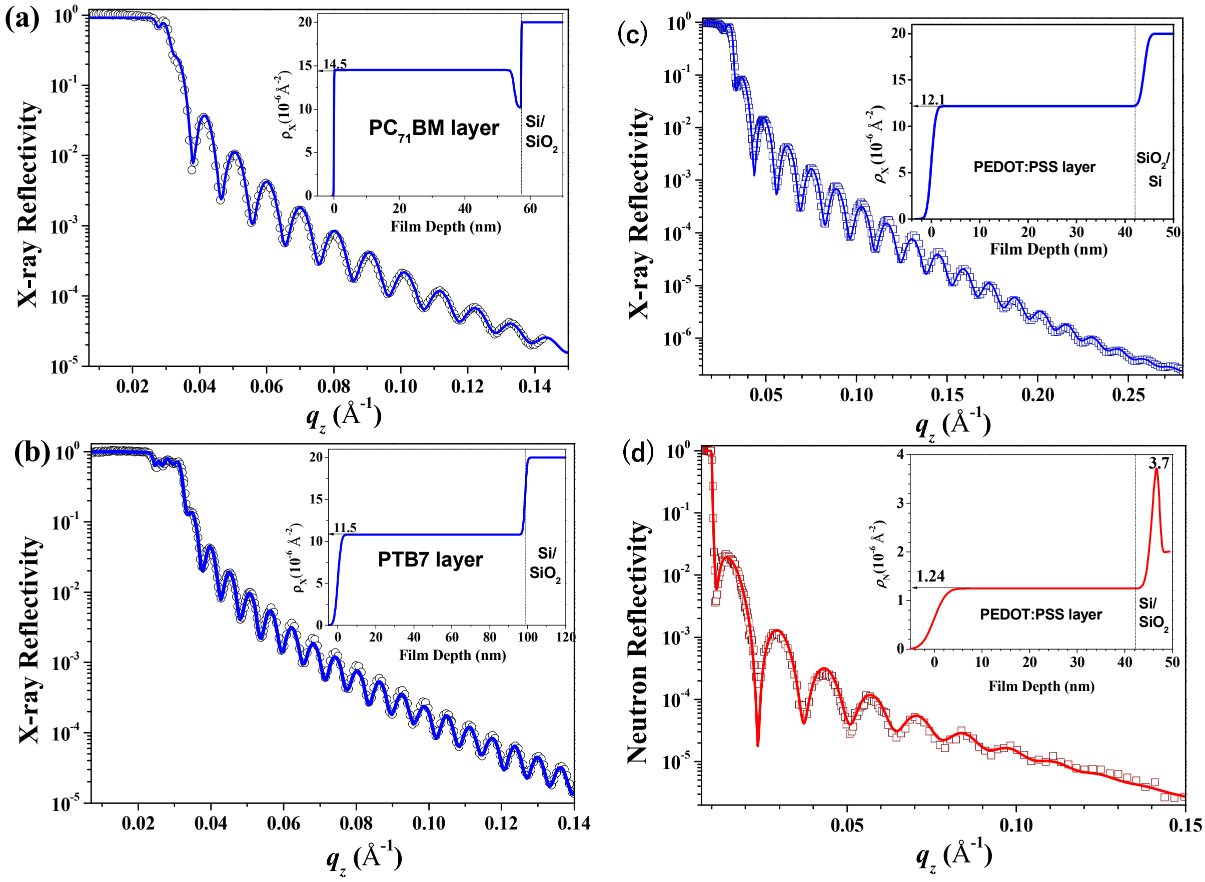

Figure S2 XR data for pure (a) $PC_{71}BM$ and (b) PTB7 films spin-cast on Si wafers. The data are fitted by single-layer model of SLD shown in the inset. The averaged SLD of the vertical SLD profiling shown in the inset is 14.5×10^{-6} Å^{-2} and 11.5×10^{-6} Å^{-2}, corresponding to averaged film density of 1.69 ± 0.1 g/cm^3 and 1.27 ± 0.08 g/cm^3 for $PC_{71}BM$ and PTB7, respectively. (c) XR and (d) NR data for the same PEPOT:PSS film spin-cast on Si wafer, with the average film SLD values indicated. From the ρ_X values of the $PC_{71}BM$ and PTB7 films, we deduced the averaged film density of 1.69 g/cm^3 and 1.27 g/cm^3, respectively. From the film densities and chemical formulae, we further deduced the ρ_N values of the neat $PC_{71}BM$ and PTB7 films to be 4.97×10^{-6} Å^{-2} and 1.16×10^{-6} Å^{-2}, respectively.

S3. XPS results

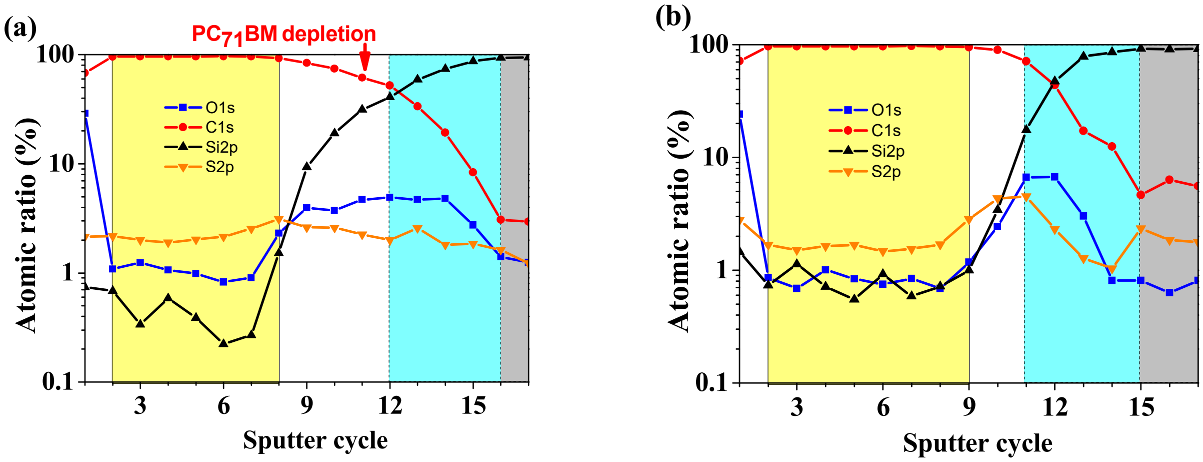

Figure S3 Through-thickness composition profiles measured with XPS (as indicated) for (a) N-1.5 and (b) D-1.5 films. Base on the etching rate (0.7 min per cycle), the yellow, cyan, and gray zones correspond mainly to the main PTB7-PC_{71}BM layer, PEDOT:PSS layer, and Si substrate, respectively. Note that the logarithmic intensity scale is used for the Y-axis to better illustrates the low contents of O1s, Si2p and S2p.

S4. GISAXS at difference beam incidence angles

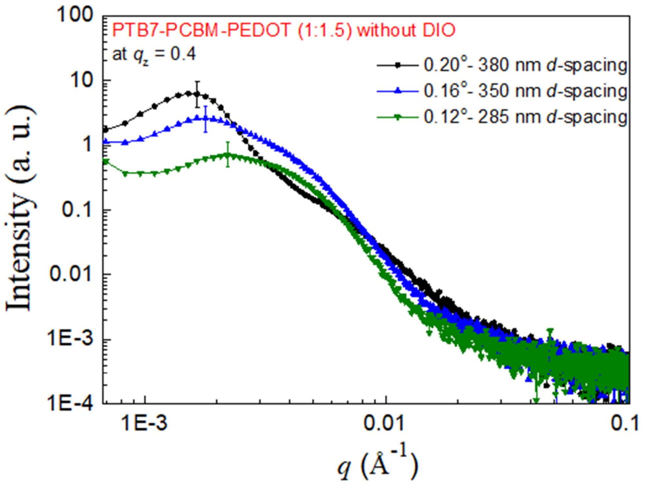

Figure S4 In-plane GISAXS profiles extracted from the 2D GISAXS patterns measured with three successive increased incident angles of 0.12°, 0.16°, and 0.2°, at q_z = 0.4 Å^{-1}. The bars mark the first hump positions of the three cases; from which, the corresponding *d*-spacing (as indicated) is deduced using the Bragg's law.

S5. GIWAXS results

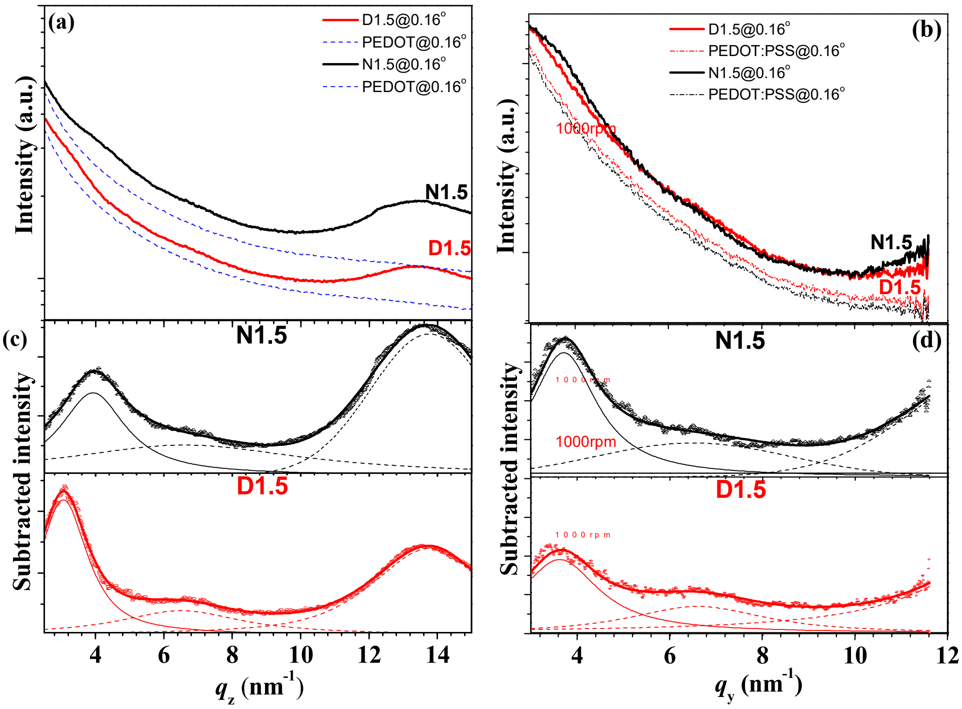

Figure S5 (a) Out-of-plane along q_z (b) and in-plane along q_y directions extracted from the GIWAXS patterns measured profiles of N-1.5, D-1.5, and the PEDO:PSS/Si films at an incident angle of 0.16°, as indicated. The corresponding (c) in-plane and (d) out-of-plane GIWAXS profiles of the N-1.5 and D-1.5 after subtracted with the scattering from the PEDOT:PSS/Si substrate. The weak PTB7 (100) peaks centered near q_0 = 3.7 nm^{-1} and the two $PC_{71}BM$ aggregate peaks centered at q_1 = 6.6 nm^{-1} and q_2 = 12.75 nm^{-1} for the two films are deconvoluted (solid and dashed curves), using the fitted parameters shown in **Table S1**. All films are spin-coated with 1000 rpm.

Table S1 Fitted parameters for the GIWAXS humps shown in **Figure S5** along in-plane q_y and out-of-plane q_z directions.

The humps are de-convoluted with Gaussian and Lorenz functions.

		PTB7 (100) packing		$PC_{71}BM$	$PC_{71}BM$
		q_0 (nm^{-1})	*d*-spacing (nm)	q_1 (nm^{-1})	q_2 (nm^{-1})
N-1.5	in-plane	3.71	1.72	6.6	13.75
	out-of-plane	3.92	1.60	6.6	13.76
D-1.5	in-plane	3.61	1.78	6.5	13.75
	out-of-plane	3.06	2.05	6.5	13.75

S6. Monte Carlo (MC) calculation

The binding energies obtained by Monte Carlo (MC) calculation can provide the energy of interaction between the specified molecules in the OPV system. The structure and partial charge of each component (molecule of CB, $PC_{71}BM$, DIO and monomer of PTB7) were first optimized via the DFT lever calculation. Using the DFT optimized structures, 10^7 configurations of each molecular pair in which the van der Waals surface of the two molecules were in contact with varied orientations were randomly generated, respectively. Moreover, the end atoms in the PTB7 monomer were set as non-contact atoms during the random sampling processes to represent the occupied space of the connected monomer in the real polymer system. The intermolecular energy for every molecular pair was determined with the DREIDING force field, which described the π-π interaction parameter of the organic/polymer systems well.[s1] The energy distribution was sampled via the calculated pair interaction energies of each configuration. Temperature effects was taken into account by weighting the distribution with the Boltzmann factor, $exp(-E_{ij}/RT)$. The average binding energy E at temperature T (with thermal energy RT) was averaged with the weighted distribution function: $<E_{ij}>_T =[\int dE\ E\ P_{ij}(E)\ exp(-E/RT)][\int dE\ P_{ij}(E)\ exp(-E/RT)]^{-1}$. Commercial package *Materials Studio* [s2] was used for DFT calculations (*Dmol*^{3}) and MC simulation engine (*Blend*).

Table S2 Calculated binding energy of either two of the four components in a CB solution containing DIO, PC71BM, and PTB7 for spin-coating, using Monte Carlo simulations.

Molecular Pair	Binding Energy (kcal/mol)
PTB7 - PTB7	-11.22
PTB7 - $PC_{71}BM$	-13.06
PTB7 - CB	-4.58
PTB7 - DIO	-4.80
$PC_{71}BM$ - $PC_{71}BM$	-13.47
$PC_{71}BM$ - CB	-5.04
$PC_{71}BM$ - DIO	-5.37
CB - CB	-2.18
CB – DIO	-2.03
DIO - DIO	-1.89

References

(s1) Mayo, S. L.; Olafson, B. D.; Goddard III, W. A. *J. Phys. Chem.* **1990**, *94*, 8897-98909.

(s2) Programs from Accelrys Inc. (San Diego, CA, USA. www.accelrys.com).

S7. In situ GISAXS over spin-casting with 0, 0.5, and 1.5 % of DIO

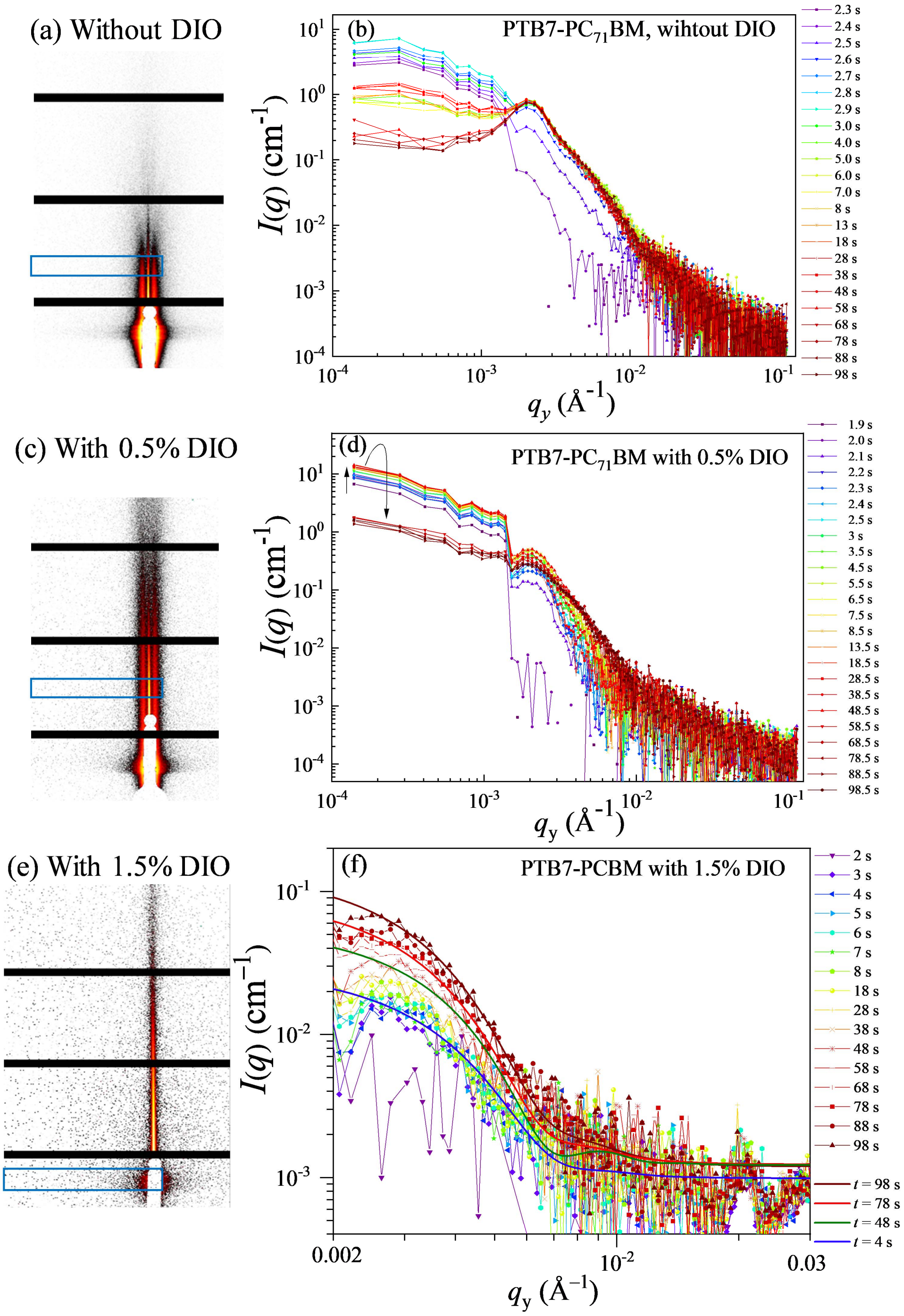

Figure S6 (a), (c), and (e) are the representative time-resolved GISAXS patterns measured after 98 s spin-coating of the CB solutions of PTB7-PC_{71}BM, added respectively with 0, 0.5%, and 1.5% (v/v) DIO. (b), (d), and (f) are the corresponding time-resolved GISAXS in-plane profiles selectively extracted at $q_z = 0.05$ Å^{-1} for the cases with (a) and (c), and at $q_z = 0.025$ Å^{-1} (for better intensity) for the case with (e) that shows no spinodal peaks (as indicated by the rectangular boxes shown). Time dependent invariant Q_{inv} values are calculated using the $I(q,t)$ profiles with the q-range displayed, and normalized by the Q_{inv} of the final GISAXS profile, as shown in Figure 9c. Data at t = 4 s, 48 s, 78 s,

and 98 s are selectively fitted (solid curves) with a sphere model of radii of 53±8 nm, 56±4 nm, 60±4 nm, and 58±3 nm, respectively. These similar sizes indicate that the GISAXS intensity growth is contributed mainly by number increase (rather than size increase) of the PCBM-rich aggregates, implying a nucleation-driven process of phase segregation.
